# *b*-Value Evaluation and Applications to Seismic Hazard Assessment

**DOI:** 10.3390/e27090958

**Published:** 2025-09-15

**Authors:** Ying Chang, Rui Wang, Peng Han, Jinhong Wang, Miao Miao, Zhiyi Zeng, Weiwei Wu, Changsheng Jiang, Lingyuan Meng, Haixia Shi, Katsumi Hattori

**Affiliations:** 1 Institute of Mining Engineering, BGRIMM Technology Group, Beijing 100160, China; changy@bgrimm.com; 2 Shenzhen Key Laboratory of Deep Offshore Oil and Gas Exploration Technology, Southern University of Science and Technology, Shenzhen 518055, China; hanp@sustech.edu.cn (P.H.); 12431098@mail.sustech.edu.cn (J.W.); zengzy@sustech.edu.cn (Z.Z.); 3 Guangdong Provincial Key Laboratory of Geophysical High-Resolution Imaging Technology, Southern University of Science and Technology, Shenzhen 518055, China; 4 Department of Earth and Space Sciences, Southern University of Science and Technology, Shenzhen 518055, China; 5 China Nuclear Power Engineering Co., Ltd., Shenzhen 518172, China; miaom@sustech.edu.cn; 6 Sichuan Earthquake Agency, Chengdu 610041, China; wwwu_seis2020@163.com; 7 Institute of Geophysics, China Earthquake Administration, Beijing 100081, China; jiangcs@cea-igp.ac.cn; 8 China Earthquake Networks Center, Beijing 100045, China; menglingyuan@seis.ac.cn (L.M.); shihaixia08@seis.ac.cn (H.S.); 9 Graduate School of Science, Chiba University, Chiba 263-8522, Japan; khattori@faculty.chiba-u.jp

**Keywords:** magnitude–frequency distribution, *b*-value, earthquake complexity, statistical seismology, stress information

## Abstract

Earthquake forecast and risk assessment are of key importance in reducing casualties and property losses. However, they have not been fully achieved due to the complexity of earthquakes. Numerous studies have explored the correspondence of the *b*-value with changes in effective stress, leveraging temporal and spatial variations to identify precursor characteristics of destructive events in both natural and induced seismic activities. However, robust interpretation of predictive *b*-values hinges on rigorous estimation, as biased results can mislead conclusions. This paper provides a comprehensive review of spatiotemporal *b*-value estimation methods alongside statistical significance tests. A pilot *b*-value analysis of natural earthquakes and induced seismicity manifested the valid impression. The expansion of monitoring datasets with the development of acquisition technology or dense array and advanced estimation methodology will augment the utility of *b*-value analysis in seismic research and hazard assessment.

## 1. Introduction

The frequency–magnitude distribution of earthquakes statistically follows the empirical power law [1,2]. The Gutenberg–Richter (GR) law has the form

(1) $$\log_{10}N\left(M\geq M_{c}\right)=a-bM,$$

where *a* and *b* are two positive constants, and $M_{c}$ is the minimum complete magnitude, above which all the magnitudes of earthquakes are completely recorded. Constant *a* describes the ambient seismicity rate of the region, and constant *b* reflects the comparative ratio of small- to large-magnitude earthquakes. Numerous studies have estimated *b*-values for various data and confirmed the universality of the GR law [3]. On a global tectonic scale, the *b*-value is ~1 for global crustal earthquakes and ~1.6 for intermediate-depth and deep earthquakes [4,5,6,7]. Induced earthquakes occurring in reservoirs, oil and gas production, waste water injection, and ${CO}_{2}$ injection also follow the GR law, typically with relatively higher *b*-values than natural earthquakes [8,9,10]. Many laboratory experiments conducted to observe the microfracturing process have found similar statistical *b*-values for microseismic events [11,12].

Although the GR relationship has been confirmed as a self-similar characteristic of natural earthquakes, induced earthquakes, and laboratory acoustic emissions, spatiotemporal variations in *b*-values have been discussed and investigated. In observational and statistical analyses, *b*-values as a function of space and time have a negative correlation with differential stress for earthquakes in the Earth’s crust and subduction zones [11,13,14,15,16,17]. Regionally, high *b*-value anomalies may correspond to high pore pressures, magmatic intrusion, and/or structural heterogeneity [18,19,20,21,22,23]. In subduction zones, high *b*-value anomalies can indicate a pathway of magma beneath volcano chains [18,24,25,26] and different physical mechanisms for the upper and lower seismic layers of double seismic zones [27,28] or deep earthquakes [29,30].

The classical estimation of spatial *b*-values is accomplished with the grid search method [31]. However, the inverse relationship between seismicity rate and grid search radius limits the spatial resolution of the *b*-value image. Another solution for spatial *b*-values uses the maximum likelihood method [28], which accommodates scattered seismic events. Data-driven penalized likelihood methods using Voronoi tessellation also provide adaptive spatial resolution and robust *b*-value estimation [32,33]. Temporal variation in *b*-values is usually affected by the successive foreshock–mainshock–aftershock sequence that lowers the magnitude of completeness in the aftershocks. One way to overcome this transient increase in the magnitude of completeness is to substitute the absolute magnitudes of the original sequence with differential magnitudes, which contain the same parameterization of the probability density distribution [34]. In this paper, we summarize spatiotemporal evaluation methods and applications.

## 2. Methods to Estimate *b*-Value

According to the GR law formula, the traditional estimation of the *b*-value involves finding the slope of the magnitude–frequency distribution in the linear logarithm regime (Equation (1)) using the least squares method. In probabilistic terms, the GR formula (1) can be expressed as the probability density function

(2) $$\mathrm{Pr}\left(M\right)=b\ln10\;e^{-b\ln10(M-M_{c})}$$

Let β=bln10; then, the probability density function (2) is

(3) $$\mathrm{Pr}\left(M\right)=\beta e^{-\beta (M-M_{c})}.$$

Then the maximum likelihood estimation is given by β=1M¯−Mc, and the *b*-value is

(4) $$b=\log_{10}e/\left(\bar{M}-M_{c}\right),$$

where $\bar{M}$ is the average magnitude of the earthquakes ($M\geq M_{c}$) [35,36]. The uncertainty in the *b*-value is estimated as one standard deviation $\sigma_{b}=\frac{b}{\sqrt{N}}$ [36] or $\sigma_{b}=2.3b^{2}\sqrt{\frac{\sum_{i=1}^{N}\left(M_{i}-\langle M\rangle \right)}{N(N-1)}}$ [37]. Marzocchi and Sandri [38] thoroughly discussed how the assumption of continuous magnitude may bias the evaluation of *b*-value and its uncertainty in the maximum likelihood estimation and provided a corrected formula to reduce the bias:

(5) $$b=\frac{1}{\ln10\left[\bar{M}-\left(M_{c}-∆M/2\right)\right]}.$$

Either the least square fitting of (Equation (1), (4) and (5)) or the maximum likelihood estimation (Equation (6)) requires an accurate assessment of $M_{c}$ to objectively interpret the *b*-value. Assuming the self-similarity of earthquakes in the catalog, an overestimate of $M_{c}$ would guarantee a *b* linear fit to the magnitude–frequency distribution but would result in a decrease in the effective number of earthquakes and a larger uncertainty of the *b*-value. Table 1 gives a summary of *b*-value estimation methods.

The magnitude of completeness is not only associated with the detectability of networks but also strongly depends on the spatial and/or temporal heterogeneity of seismicity. Usually, once the network facilities are built, the detectability of the network is determined. With the assumption of self-similar earthquakes, $M_{c}$ can be evaluated by the magnitude with the highest frequency (maximum curvature) or by scanning the fitness of the GR relationship (for example, with the goodness-of-fit test [39]) and $M_{c}$ with its *b*-value stability [40]. Woessner and Wiemer [41] proposed the entire-magnitude-range (EMR) method, which inverts the detection capability of the incomplete part for *a-* and *b*-values of the complete subset by solving a maximum likelihood estimator. Compared to other methods, the EMR method is more stable but requires the most computational consumption. Mignan and Woessner [42] summarized methods of assessing $M_{c}$. Later, Lombardi [43] and Taroni [44] offered a better estimate of $M_{c}$ using statistical methods. Where the catalog is incomplete, such as aftershock sequences and mining-induced seismicity contaminated by quarry and blast, *b*-value analysis may be beneficial in identifying the incomplete catalog [21,38,41,45,46]. Therefore, the *b*-value and its uncertainty should be carefully assessed before making an inductive judgment regarding *b*-variation.

**Table 1:** Summary of *b*-value estimation methods.

Method	Principle	Advantages	Limitations	Applicable Scenarios
Least Squares Method [1,2]	Best-fit line slope for earthquake distribution (minimizing distance)	Simple and straightforward	Easily biased by large earthquakes [47]	Relatively complete catalogs
Maximum Likelihood Estimation [35,36,38]	Best-fit distribution for earthquake (maximum likelihood function)	Not affected by specific large earthquake	Strongly dependent on the accurate assessment of $M_{c}$	Relatively complete catalogs
B-Positive Estimator [34]	Estimation of temporal *b*-values of magnitude difference following the Laplace distribution	Robust, insensitive to changes in detection rate	Relies on positive magnitude difference	Temporal sequence with mainshocks
Classical Grid Search Method [31]	Search for events in nearby grid points to calculate *b*-value	Simple and straightforward	Requires a minimum sample size	Catalog including spatial and temporal heterogeneity
Objective Bayesian Method [28]	Addition of spatial/temporal derivative penalties to the likelihood function	High spatial resolution and coverage	Relatively complex calculation	Catalog including spatial and temporal heterogeneity
Data-Driven Method [33]	Average of top models from randomly generated models	Adaptive and objective	Complex calculation and computational consumption	Catalog including spatial and temporal heterogeneity

### 2.1. B-Positive Estimator of b-Values for Aftershock Sequences

Temporal variation in *b*-values was validated to determine the mainshock in a foreshock–mainshock–aftershock sequence. In real-time observation, a large earthquake would affect the detection of small earthquakes and increase the magnitude of completeness; therefore, the *b*-value of the increased incomplete sequence appears biased to decrease. To overcome the incompleteness magnitude, van der Elst [34] proposed a new estimator of *b*-values using the distribution of magnitude differences between successive earthquakes. Given that the magnitudes of earthquakes follow the exponential distribution (Equation (3)), the Laplace distribution [48] gives the same estimator when substituting the magnitudes with magnitude differences (Equation (6)).

(6) $$\mathrm{Pr}\left(\left|m^{\prime }\right|\right)=\beta e^{-\beta \left|m^{\prime }\right|},$$

where $m^{\prime }$ is the magnitude difference of successive earthquakes. For the Laplace distribution, the β parameter of the maximum likelihood estimation is =1m′¯. The estimator can be independently calculated by either positive or negative magnitude differences. A big earthquake temporarily blocks the detection of weaker earthquakes, resulting in a negative successive magnitude difference in an aftershock sequence. The estimator based on positive magnitude differences (*b*-positive) produces a robust estimation of *b*-value (Equation (7)) [34], even if positive magnitude differences are not significantly impacted by the detection rate.

(7) $$\beta^{+}={\left(\bar{m^{\prime }}-M_{c}^{\prime }\right)}^{-1},\;m^{\prime }\geq M_{c}^{\prime }\;,$$

where $M_{c}^{\prime }$ is a minimum magnitude difference.

### 2.2. Classical Grid Search Method

The classical method of computing spatial *b*-value variations is the grid search method in the ZMAP software package, which grids the study area and searches a certain radius or a specific number of closest earthquakes by centering the grid node and then estimates the complete magnitude and *b*-value at grids from extracted subsets [31]. The advantage of ZMAP is that it explicitly expresses the GR law and the uncertainty of the results, but it requires the minimum number of earthquakes in the calculation.

The significance of *b*-values in two different groups can be statistically assessed using the Utsu test [49],

(8) $$∆AIC=-2\left(N_{1}+N_{2}\right)\ln\left(N_{1}+N_{2}\right)+2N_{1}\ln\left(N_{1}+N_{2}b_{1}/b_{2}\right)+2N_{2}\ln\left(N_{2}+N_{1}b_{2}/b_{1}\right)-2,$$

where $N_{1}$ and $N_{2}$ are the numbers of events in two sample sets, and $b_{1}$ and $b_{2}$ are *b*-values in two samples. The probability of accepting the null hypothesis $b_{1}$ = $b_{2}$ is defined as $p=exp\left(-∆AIC/2-2\right)$. If ∆AIC≥2, it usually means that the two *b*-values are affirmably different. When ∆AIC>5, the difference in *b*-values is highly significant.

### 2.3. Objective Bayesian Method

Another approach is to statistically treat earthquakes as marked point processes. This method constructs functional parameters of spatial *b*-values and solves the maximum of the optimal posterior function [28], which is included in the software package for hierarchical space–time point-process models (HIST-PPM) [28]. The maximum likelihood method has a low computational cost and is stable for small sample numbers of events [41,50,51].

The likelihood function is

(9) $$L\left(\beta \right)=\prod_{i=1}^{N}\beta e^{-\beta (M_{i}-M_{c})},M_{i}\geq M_{c},$$

Assuming that the positive *b*-value is a function of the earthquake locations, β(x,y) is fulfilled as $\beta \left(x,y\right)=e^{\theta (x,y)}/\log_{10}e$. Spatial gridding is implemented by the Delaunay tessellation, and the vertices of the Delaunay triangulation are the earthquake locations [52]. In the Delaunay tessellation, θ(x,y) is a piecewise linear function of the three vertices inside one triangle.

It is conventional to maximize the log of the likelihood function for computational efficiency and to prevent overflow.

(10) $$\ln L\left(\theta \right)=\sum_{i=1}^{N}\left\{\theta \left(x_{i},y_{i}\right)-e^{\theta \left(x_{i},y_{i}\right)}\left(M_{i}-M_{c}\right)/\log_{10}e\right\}-N\ln\log e,{\;M}_{i}\geq M_{c}$$

The parameterized function $\theta \left(x_{i},y_{i}\right)$ of *b*-values should not only produce a good fit to the data but also be smooth without abrupt local variation. A measure of the rapid local variation is given by a roughness penalty as follows:

(11) $$R\left(\theta |w\right)=\ln L\left(\theta \right)-w\iint \left\{{\left(\frac{\partial \theta \left(x,y\right)}{\partial x}\right)}^{2}+{\left(\frac{\partial \theta \left(x,y\right)}{\partial y}\right)}^{2}\right\}dx\;dy$$

where w is the penalty weight. The maximum log-likelihood and its maximizing parameter θ are computed by the objective Bayesian method [53]. The optimal penalty weight is objectively chosen according to Akaike’s Bayesian Information Criterion (ABIC) [54].

### 2.4. Data-Driven Method

The third *b*-value imaging method is data-driven approach to determining the heterogeneity of the model. The complexity of the spatial *b*-value is controlled by the number of Voronoi tessellation cells. A certain number of Voronoi seeds are randomly replaced. The best-fitting model of spatial *b*-values is found from comparing the homogenous model to various partitioning models.

This method utilizes Voronoi tessellation to minimize parameterization and selects the best-performing models according to the Bayesian Information Criterion for all random realizations [32,55]. Si and Jiang [55]’s method estimates *b*-values based on the detection rate function of Ogata and Katsura [56], whereas Kamer and Hiemer (2015) directly calculated *b*-values for discretized data using the formula of Tinti and Mulargia [57]

(12) $$b=\frac{1}{\ln\left(10\right)∆m}\ln\left(1+\frac{∆m}{\bar{M}-M_{c}}\right)$$

where ∆m is the discrete magnitude distance. First, a certain number *N* of random nodes separates the study region into *N* Voronoi cells. Assuming parameters are segment-linearized at the Voronoi vertices, the model is constructed with *N* sets of parameters’ *b*-values (Equation (11)) or earthquake detection rate and range (Equation (5) in [55]). Finally, the optimal parameters are selected using the Bayesian Information Criterion (BIC) [58],

(13) $$BIC=-\ln L+\frac{k}{2}\ln N$$

where *L* is the likelihood function of one model (Equation (9)), *k* is the degrees of freedom of one model, and *N* is the number of data points.

The data-driven method also extends to evaluating time series of *b*-values [59,60,61,62,63,64,65]. The significance and change points of temporal *b*-value variations are scalable by the Bayesian method through numerous random models with a specific number of change points in *b*-values.

## 3. Applications of Spatiotemporal *b*-Value Estimation

**Figure 1:**
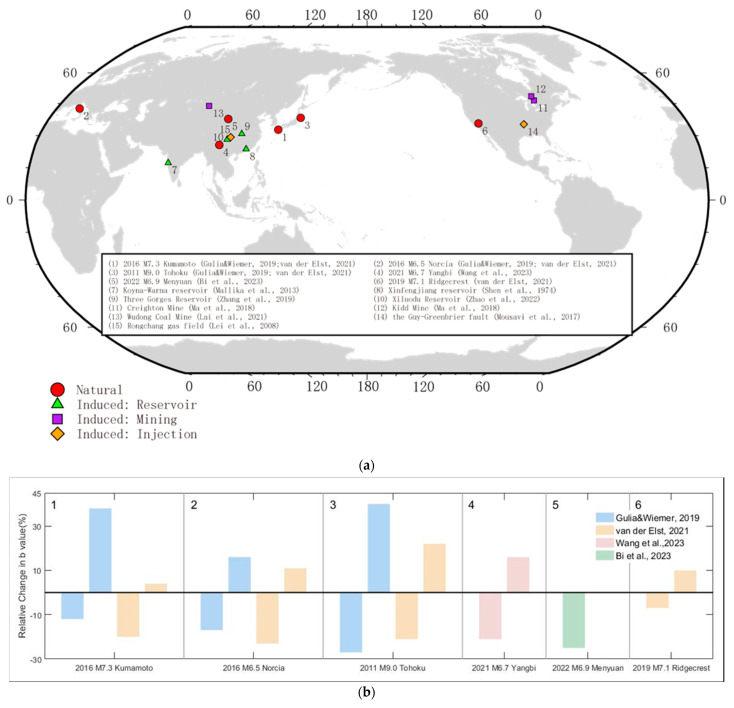

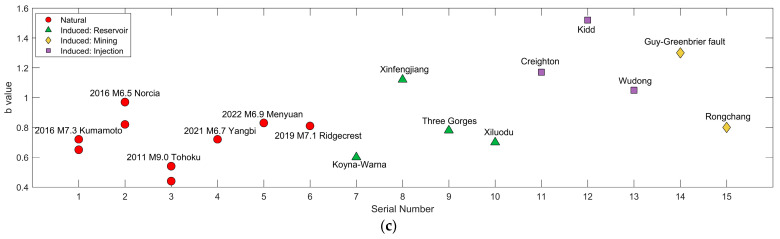
(**a**) A total of 15 *b*-value analysis cases are plotted in the global map; the studied cases are labeled in serial number [23,34,51,61,68,76,79,80,81,82,83,84]. (**b**) *b*-value changes in six major foreshock–mainshock–aftershock sequences estimated using the traffic light system; a negative *b*-value change is the precursor of the mainshock, while a positive *b*-value change indicates a strong aftershock [34,51,61,74]. (**c**) *b*-values before mainshock or rockburst from the case studies in (**a**) are plotted to show a case-by-case variation. The *b*-values of natural sequence 1–3 are from two references.

The applications of *b*-values for forecasting large earthquakes and monitoring induced seismicity are summarized in this paper (Table 2). Taking advantage of the inverse relationship between *b*-values and differential stress, the spatiotemporal distribution of *b*-values sheds light on the evolution of stress in seismogenic zones and helps to anticipate seismic risk [13,66,67,68,69]. A temporal decrease in the *b*-value before a great earthquake was observed in the 2003 Tokachi-Oki earthquake ($M_{w}$8.3) [70], the 2004 Sumatra earthquake ($M_{w}$9.1) [71,72], the 2008 $M_{w}$7.9 Wenchuan earthquake [73,74,75], the 2011 $M_{w}$9.0 Tohoku earthquake [71], the 2014 *M*7.3 Kumamoto earthquake [76,77], the 2019 Ridgecrest earthquake [34,66,78], and the assessment of the earthquake forecast in Yunnan, China. Therefore, decreasing *b*-values can be a precursor of impending large earthquakes (Figure 1).

**Table 2:** Applications of spatiotemporal *b*-value estimation.

Category	Key Applications	*b*-Value Characteristics	Reference
Foreshock–mainshock sequence	Real-time discrimination of sequence	*b*-value decrease before mainshock	[34,61,71,72,74,76,78,85,86]
Medium-to-long-term changes before large earthquakes	Stress concentration assessment in seismogenic zones, identification of potential nucleation zones	Significant *b*-value decrease near future epicenters pre-mainshock, low *b*-value zones in high-risk regions	[51,66,71,78,87,88,89,90,91,92,93,94,95,96]
Injection-triggered earthquakes	Real-time monitoring of injection impacts, early warning of small-to-moderate induced events	Higher *b*-values compared to natural earthquakes, *b*-value drop prior to main events and then rebounds, spatial heterogeneity linked to fault reactivation	[60,97,98,99,100,101,102,103,104,105,106,107,108]
Water reservoir-induced earthquakes	Assessing seismic response to impoundment/water level fluctuations	Slightly higher *b*-values than natural earthquakes, elevated *b*-value during initial impoundment	[79,80,81,82,109,110,111,112,113,114,115,116,117,118]
Mining-induced microseismicity	In situ stress evaluation, seismic hazard analyses	Generally higher *b*-values than natural earthquakes, *b*-value decreases before rockbursts	[8,45,46,83,96,119]

### 3.1. Natural Earthquakes

#### 3.1.1. Foreshock–Mainshock Sequence

Real-time discrimination of a foreshock–mainshock–aftershock sequence is important for seismic hazard mitigation. Retrospective analyses of historical mainshock sequences have confirmed consistent variations in *b*-values before a great failure [13,34,74,76,78] (Figure 1), and laboratory experiments have also shown this [11,16,17].

After a meta-analysis of 37 foreshock studies, Mignan [85] confirmed that the foreshock–mainshock pattern commonly exists and is valuable for earthquake prediction as long as small-magnitude events are considered relative to the mainshocks. Gulia and Wiemer [76] proposed a traffic light system to real-time discriminate between foreshocks and aftershocks. The discrimination criterion is based on real-time changes in *b*-values. Strong aftershocks could cause severe damage and jeopardize the mainshock rescue. The precursory sign of *b*-value decrease commonly exists in the 2016 Amatrice-Norcia foreshock–mainshock sequence, the 2016 Kumamoto earthquake sequence, and the 2011 Tohoku earthquake sequence [34,76,86] (Figure 1b). To mitigate aftershock risk, Bi, Yin, Jiang, Yin, Ma and Song [61] further proposed a robust aftershock traffic light system based on the distinctions between the *b*-values of aftershock sequences and background events. Since a large earthquake will raise the magnitude of completeness and temporarily confuse the temporal *b*-value estimate, the *b*-positive estimator provides a robust temporal measurement of the *b*-value [34].

The initial traffic light system uses a threshold according to the empirical evaluation of the historical earthquake sequence. Robust and objective criteria of *b*-value change are needed. An optimization of the traffic light system with Akaike Information Criterion (AIC) and bootstrap instead utilized the significance level of the difference in *b*-values between the sample and the background rather than an arbitrary constant to control traffic light signals [86]. The optimized traffic light system explicitly recognized the foreshock–mainshock–aftershock sequence of the 2021 Yangbi earthquake through temporal and spatial variations in *b*-values (Figure 1b).

#### 3.1.2. Medium-to-Long-Term b-Value Change Before Large Earthquakes

Numerous studies have investigated the spatiotemporal evolution of the *b*-value prior to large earthquakes, consistently revealing that a significant decrease in *b*-value often occurs near the future epicenter in the lead-up to mainshock events [78,87,88,89,90,91,92,93]. This phenomenon has been widely interpreted as a manifestation of stress concentration during the nucleation phase of a large earthquake [11,120,121] and thus has been broadly applied in medium-to-long-term seismic hazard assessment, with its effectiveness validated in multiple major earthquake cases.

Approximately three years before the 2008 Wenchuan earthquake ($M_{s}8.0$), the regional *b*-value began a gradual decline, followed by a rapid decrease during the latest six months prior to the mainshock. Notably, the spatial distribution of a low *b*-value zone closely coincided with the actual rupture area [74]. Similarly, in the case of the 2011 Tohoku-Oki earthquake ($M_{w}9.0)$ in Japan, the *b*-value began to gradually decrease since 2007, with the declining trend persisting for over four years [71]. Before the 2004 $M_{w}9.0$ Sumatra earthquake, the *b*-value in the epicentral region was consistently low (1.0~1.2) for a long time, and the region closest to the mainshock epicenter had the largest decline in *b*-value, with a localized loss of up to 40% [71,122]. In addition, prior to the 2016 Kumamoto earthquake ($M_{w}7.3$) and the 2019 Ridgecrest earthquake ($M_{W}7.1$), localized low *b*-value zones developed along the main fault traces. It is noteworthy that the low *b*-value zone of the Ridgecrest earthquake showed a temporal movement in the direction of the ultimate epicenter [66,94,123,124]. Nonetheless, the applicability of the *b*-value in earthquake forecasting remains in debate. For instance, for the case of the 2011 Van–Erciş earthquake in Turkey ($M_{w}7.2$), some studies reported a significant decrease in the *b*-value from 1.7 to 0.6 within one year prior to the mainshock, with the low-*b*-value zone highly consistent with the eventual hypocenter, supporting its interpretation as a potential precursor [95,125]. In contrast, Kamer and Hiemer [126] contended that this apparent trend was primarily an artifact of the sliding time window method, which included the mainshock and its aftershocks, thereby questioning its predictive validity. Therefore, restricted analysis and statistical assessment of *b*-value change are necessary for the precursory evaluation.

**Figure 2:**
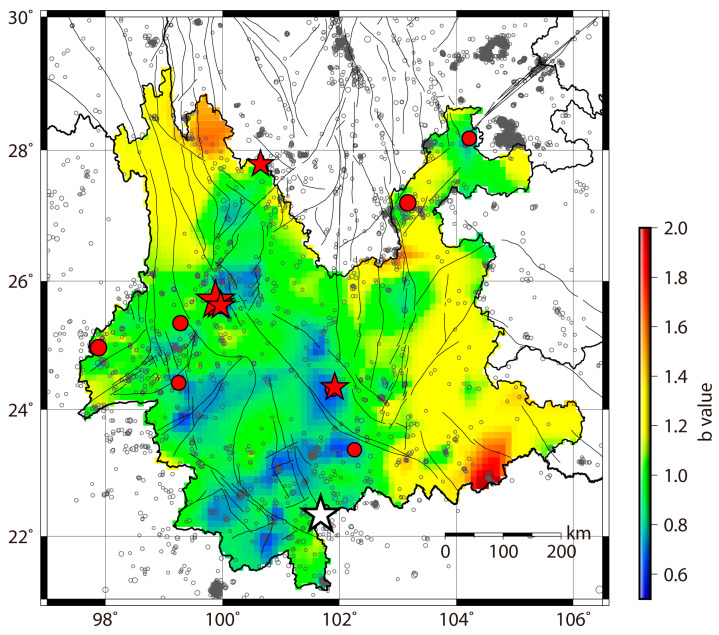
*b*-values from January 2015 to December 2019 earthquake forecasts with M ≥ 5.0 from January 2020 to December 2024 in Yunnan, China (Figure 4a in [51]). The gray dots are background earthquakes. The red dots and red stars scaled to the magnitude represent earthquakes with M ≥ 5.0 and M ≥ 5.5 that occurred from January 2020 to December 2024.

Regional studies in continental regions with high earthquake risk provide additional evidence for the *b*-value’s predictive power. Hu, Han, Wang, Shi, Chen and Li [96] conducted a systematic analysis of the earthquake catalog from 1970 to 2023 at the northeastern margin of the Tibetan Plateau using the HIST-PPM method. Significant spatial variation in *b*-values was found throughout the region, with low *b*-value zones primarily forming along the Zhongwei Fault and the West Qinling Fault Zone. These zones exhibit a strong spatial correlation with the distribution of historical moderate-to-large earthquakes, thereby reinforcing the validity of the *b*-value as a robust medium-to-long-term indicator for identifying potential seismogenic zones. Wang, Chang, Miao, Zeng, Chen, Shi, Li, Liu, Su and Han [51] conducted a stage-wise spatiotemporal analysis of *b*-value evolution in the Yunnan region and found that low *b*-value zones remained spatially stable over time, with a significant concentration of moderate-to-large earthquakes within these zones (Figure 2). The spatial *b*-value threshold of forecasting should be cautiously analyzed based on background seismicity and in situ stress status because the different tectonic settings compose relative spatial low *b*-value patterns due to the negative linear relation between *b*-value and differential stress [13]. An optimal *b*-value threshold applicable to regional forecasting was explicitly defined using Molchan error diagrams and probabilistic gain metrics [127]. This provides a more practical and quantitative standard for applying *b*-value analysis in medium-to-long-term earthquake forecasting within complex fault systems. Overall, the spatiotemporal evolution of the *b*-value using statistical seismological methods has become one of the key approaches for identifying potential nucleation zones of large earthquakes. As earthquake catalogs improve in magnitude completeness and spatiotemporal resolution, the application of *b*-value analysis in pre-earthquake identification is gaining traction.

### 3.2. Induced Seismicity

#### 3.2.1. Injection-Triggered Earthquakes

For tectonic earthquakes, the *b*-value typically ranges between 0.8 and 1.2. In contrast, higher *b*-values (b>1.5) are commonly observed in seismicity induced by unconventional gas exploration, suggesting that a magnitude distribution is more dominated by smaller events compared to typical tectonic earthquakes [22,23,107,128,129,130]. The *b*-value of induced seismicity not only indicates changes in rupture scale and stress conditions but also acts as an important physical parameter for real-time seismic monitoring and early warning of small to moderate-magnitude earthquakes. Its extensive application in unconventional natural gas production projects has proven its efficacy.

In the United States, particularly in Texas, where shale gas production is extensive, seismicity typically has high *b*-values (generally above 1.2), indicating that fluid injection primarily induces microseismic events [100,108] (Figure 1c). Near the ancient fault Azle zone, studies have shown that *b*-values significantly decrease to 0.7~0.8 prior to the occurrence of moderate earthquakes (M≥4.0) and then rapidly rebound afterward, corresponding to brine production activity [100]. The seismicity in the Guy–Greenbrier area, Arkansas, is correlated with pore pressure change, as the spatiotemporal evolution of *b*-values indicates [68].

In British Columbia, seismicity features vary significantly between two main induced seismic activity zones: the northern Montney region and the Kiskatinaw area south of Fort St. John [97,99,104]. In the northern Montney region, *b*-values range from approximately 1.0 to 1.3, and seismic events often exhibit a delayed response of up to two days following the completion of hydraulic fracturing operations. This implies that delayed pore pressure diffusion or far-field stress perturbations may have an impact on the earthquakes. On the other hand, the Kiskatinaw area has higher *b*-values of around 1.5, indicating a dominance of small-magnitude events, the majority of which occurred during injection or soon after the end of fracturing operations [97,98,99].

In the southern Sichuan Basin, China, the Rongchang, Changning, and Weiyuan shale gas production zones exhibit pronounced spatial heterogeneity and temporal evolution in *b*-values. Based on a catalog of over 240,000 events observed by a dense seismic array, Huang, et al. [131] reported that the average *b*-value in the Changning area is 1.06, which is higher than the regional background level in Sichuan Province (~0.8), but there is noticeable spatial heterogeneity. Hu, Xu, Chen, Zhang, Cao and Wang [106] found that the overall *b*-value in the Changning–Zhaotong shale gas projection zone is 0.98, exhibiting a clear bilinear fitting pattern and offset spatial distribution. These features reflect the diversity of seismogenic environments and the high complexity of the region’s response to stress perturbations. Specifically, the *b*-value in the Changning–Shuanghe salt mining area reaches as high as 1.25. Moreover, most M≥4.0 earthquakes are concentrated in the low-*b*-value regions. Based on a spatiotemporal analysis of microseismic data, Jiang, Han, Long, Lai, Yin, Bi and Si [60] employed a non-parametric approach to identify a low-*b*-value anomaly near the epicenter of the 2019 Changning $M_{S}6.0$ earthquake, with its spatial extent roughly corresponding to the source rupture area. This finding further supports the validity and effectiveness of the *b*-value as a physical indicator for delineating the potential rupture zone and regions of high stress concentration. Further analysis of temporal evolution revealed that several earthquake clusters exhibited a significant drop in *b*-value prior to moderate events, followed by a rapid rebound afterward, reflecting a stress accumulation and release process similar to that of tectonic earthquakes [102]. These findings suggest that the *b*-value holds promise for the early identification of induced moderate earthquakes. Related studies have further validated the sensitivity and applicability of *b*-values in this region from multiple perspectives [105,106].

In the Weiyuan area, *b*-values from a high-precision earthquake catalog based on 18,663 seismic events show systematic increase with depth, indicating a dominance of small-magnitude events at greater depths [103]. In contrast, Chen, Meng, Niu, Tang, Yin and Wu [101] observed extremely low *b*-values (approximately 0.47) farther from wellbore, which are primarily associated with natural fracture, whereas events near the wellbore tend to have relatively higher *b*-values. This further highlights the potential of the *b*-value as a key physical parameter for distinguishing between fault-controlled and injection-dominated seismicity. Moreover, it can be applied to real-time monitoring and operational decision making [132].

#### 3.2.2. Water Reservoir-Induced Earthquakes

In reservoir-induced seismicity, the *b*-value typically exhibits slightly higher or temporarily elevated trends compared to tectonic earthquakes, reflecting weaker stress concentration and limited rupture scales in the source region [112,133]. Numerous studies have shown that during the initial impoundment phase and periods of rapid water level fluctuations, the regional *b*-value often increases significantly, accompanied by enhanced small-magnitude seismic activity.

The Koyna Reservoir in India represents one of the most well-documented cases of reservoir-induced seismicity. Prior to the *M*6.3 mainshock in 1967, the *b*-value of the regional foreshock sequence rose to approximately 1.2, significantly higher than the Indian Peninsula’s average of 0.47. Following the mainshock, the *b*-value of the aftershock sequence declined to around 0.85~1.0 but remained elevated compared to typical values in stable continental regions [111,112,113,114,115,133]. These observations indicate that stress release in the region was dominated by small-magnitude events, and that variations in the *b*-value may possess potential precursory signature. Subsequent studies further confirmed that the *b*-value in the Koyna–Warna region generally exceeds 1.0, reinforcing the recognition of elevated *b*-values as a key statistical characteristic of reservoir-induced seismicity [79,109,110].

In China, numerous studies have confirmed that the spatiotemporal evolution of *b*-values in reservoir-induced seismicity exhibits strong regional characteristics. The Xinfengjiang Reservoir is one of the earliest documented cases of strong reservoir-induced earthquakes. Prior to the 1962 M6.2 earthquake, the *b*-value of the foreshock sequence was 1.12, significantly higher than the regional average of 0.72 during the same period [80]. After the mainshock, the *b*-value decreased to 1.04. Meanwhile, the focal depth shifted from 1~4 km during the foreshock stage to 4~7 km in the aftershock stage, revealing a consistent spatiotemporal and statistical pattern in reservoir-induced seismicity. Since the impoundment of the Three Gorges Reservoir began in May 2003, the surrounding region has been seismically active. Zhang, Lei, Liao, Li and Yao [81] analyzed 3,400 seismic events with M ≥ 0.7 recorded between 2003 and 2017 and obtained an overall *b*-value of approximately 0.97 using the maximum likelihood estimation method. This value is higher than the regional tectonic background *b*-value, which ranges from about 0.6 to 0.8. Further, high *b*-values of 0.96, 1.14, and 0.94 corresponding to three typical water level stages of 135 m, 156 m, and 175 m, respectively, indicate a high proportion of small-magnitude events and the dominance of induced seismicity mechanisms [118]. After the impoundment of the Zipingpu Reservoir, located near the Wenchuan fault zone, a large number of shallow microseismic events occurred. Although no significant decrease in *b*-value was observed prior to the Wenchuan earthquake, the increased density of microseismic activity and the fluctuating trend in *b*-values suggest that the reservoir exerted a sustained perturbation on the shallow stress field in the source region [117].

Along the Jinsha River, the *b*-value of seismicity exhibits pronounced spatiotemporal heterogeneity due to the combined effects of multiple reservoir operations and complex tectonic settings. The earthquakes near the Xiluodu dam are primarily located in shallow limestone formations, with focal depths generally less than 5 km, and the *b*-value is around 1.2 [82]. The *b*-value of the earthquake sequence associated with the $M_{S}5.2$ event that occurred in 2014 near Wuji Town, downstream of the Xiluodu Dam, was relatively low at approximately 0.7. Overall, hydrological disturbances induced by reservoir operations intensify the heterogeneous evolution of the subsurface stress field, and the spatial distribution of *b*-value can serve as an important indicator for identifying high-risk zones of reservoir-induced seismicity and regions of localized stress concentration.

#### 3.2.3. Microseismicity in Mines

Seismic hazard analyses indicate that mining-induced seismicity follows the same magnitude–frequency relationship as natural tectonic earthquakes; however, the *b*-value of mine quarry and blast-contaminated events is generally higher. This characteristic is of great significance for the efficient and quantitative assessment of seismic hazards in underground mines [46,119]. In the context of underground mining, *b*-value analysis has become a widely recognized tool for evaluating in situ stress levels and forecasting microseismic hazards.

In the Canadian Creighton and Kidd mines, *b*-values of multiple mining-induced seismic sequences showed a declining trend before the mainshock, dropping to as low as ~0.8 before quickly rising to a peak around the mainshock (e.g., reaching 1.52 during the 2011 mainshock at the Kidd mine) [83]. These findings demonstrate the usefulness of the *b*-value as a potential hazard index for mining-induced seismic events. Wang, Wu, Liu and Li [8] also found a strong correlation between the evolution of the *b*-value and the rock mass failure process at the 8^#^ orebody of the Huize lead–zinc mine in Yunnan Province. Mining excavation induces disturbance and even localized stress concentrations in the rock mass, which may promote crack compaction, initiation, and propagation. During this phase, the *b*-value generally shows an increasing trend. A considerable decrease in the *b*-value, on the other hand, signals the beginning of rock mass instability and a noticeably higher failure likelihood [84]. Palgunadi, Poiata, Kinscher, Bernard, De Santis and Contrucci [45] applied an adapted full-waveform-based automatic method in the Garpenberg mine, Sweden, which greatly improved the identification and location of microseismic events. They found that the *b*-value for production blast-induced events was around 1.8, while non-rupture events from mechanical noise reached 4~5. This highlights the usefulness of *b*-value analysis for source-type discrimination and noise filtering, enhancing the reliability of seismic monitoring in mines.

In a regional comparative study, Hu, Han, Wang, Shi, Chen and Li [96] analyzed seismic activity in the Huating and Tianzhu areas of China, both exhibiting high *b*-values exceeding ~1.5. By introducing the day-to-night event ratio (D/N), they found that mining methods were closely linked to seismic patterns. Huating, dominated by 24-hour coal mining, showed a balanced D/N of 0.97, while Tianzhu, where daytime blasting is used for limestone extraction, had a D/N of 3.72, indicating daytime-concentrated activity. These contrasts suggest that operational schedules significantly affect seismicity and the distribution of *b*-values. Although numerous studies support the precursor potential of *b*-values, their predictive reliability remains limited. *b*-values are sensitive to catalog completeness, location uncertainty, and sampling window and are further influenced by orebody geometry and anthropogenic disturbances. Therefore, interpreting the spatial and temporal evolution of *b*-value requires integration with site-specific geomechanical models and mining methods.

## 4. Discussion and Conclusions

The development of innovative distributed acoustic sensing technology and the cost-efficient installation of nodal seismic arrays certainly benefit earthquake monitoring for detection of small-magnitude events and catalog completion [134,135]. With the surge of waveform records and the revolutionizing application of powerful machine learning, earthquake detection and processing have not only been quantitatively advanced and made more efficient; they have also expanded beyond the small-magnitude limitation [136,137,138]. The application of advanced acquisition technologies and seismic processing techniques has laid the foundation for *b*-value analysis.

Rigorous *b*-value analysis methods ensure the usability of *b*-values as an indicator of large events. The *b*-positive estimator uses positive differences between successive earthquake magnitudes of an incomplete catalog, proving robust to detection gaps following large earthquakes. Spatial variations in *b*-value are analyzed via grid search methods (such as ZMAP), with significance tested using the Utsu method. The objective Bayesian method models the *b*-value as a spatial function using Delaunay tessellation, optimizing fit while minimizing abrupt local variations. Data-driven approaches use Voronoi tessellation and the Bayesian Information Criterion to select optimal models, extending to temporal changes by identifying significant shifts through random model testing. These methods balance accuracy, computational cost, and adaptability to catalog completeness and variability.

The Gutenberg–Richter *b*-value as a negative associator of differential stress has been widely used in seismic hazard probability assessment. However, the spatial–temporal *b*-value estimation is dependent on the magnitude completeness and the statistical stability of seismicity. Therefore, *b*-values should not be biased estimates and the significance of *b*-value differences should be evaluated before further interpretation [139].

A significant perturbation in the *b*-value can indicate a change in stress or a more complex, non-linear dynamic process. This perturbation can be a key piece of information about the complexity of a fault system. The characteristic *b*-value decrease before mainshocks in natural foreshock–mainshock–aftershock sequences aids in real-time discrimination of mainshocks via traffic light systems optimized with AIC. Although debates exist, in the medium to long term, pre-mainshock *b*-value drops near epicenters support hazard assessment. Induced seismicity generally shows distinct higher *b*-value patterns than tectonic earthquakes associated with well injection, reservoir impoundment, or mining projection. Still, spatiotemporal *b*-value analysis of induced or triggered seismicity helps assess in situ stress and operational impacts. When integrated with geological context, it enhances forecasting accuracy and operational decision making, making *b*-values a versatile tool for seismic risk management.

In conclusion, advancements in data acquisition and quality expanding the limitation of the magnitude completeness will provide plenty of databases for *b*-value analysis. The robustness and resolution of *b*-value estimation are committed by innovative methodology in conjunction with critical analysis and discrimination. Integrated with cross-disciplinary interpretation, the *b*-value is a strong precursor, particularly in distinguishing foreshocks from background seismicity, refining medium-to-long-term hazard maps, identifying rupture zones, assessing seismic hazard mitigation, and providing quantitative risk warning of rock mass.

## Data Availability

No new data were created or analyzed in this study. Data sharing is not applicable to this article.
